# Incidence and histological patterns of thyroid cancer in Sri Lanka 2001-2010: an analysis of national cancer registry data

**DOI:** 10.1186/s12885-018-4083-5

**Published:** 2018-02-07

**Authors:** Umesh Jayarajah, Ashan Fernando, Saumyakala Prabashani, Eshani A. Fernando, Sanjeewa A. Seneviratne

**Affiliations:** 1 Department of Surgery, Faculty of Medicine, University of Colombo, Colombo, Sri Lanka; 2 National Cancer Control Programme, Ministry of Health, Colombo, Sri Lanka

**Keywords:** Thyroid cancer, Incidence, Papillary cancer

## Abstract

**Background:**

An increasing incidence of thyroid cancer is observed in many developed countries. Increasing incidence may also reflect better reporting or increased diagnostic scrutiny. We conducted this study to examine trends in thyroid cancer incidence and histological patterns in Sri Lanka.

**Methods:**

A retrospective cohort evaluation of patients with thyroid cancer during 2001–2010 was performed using population based data published from the Sri Lanka National Cancer Registry. Trends in incidence and histological patterns were analysed by age and gender.

**Results:**

The age-standardized incidence of thyroid cancer increased from 2.44 per 100,000 in 2001 (95% confidence interval [95% CI]: 2.21–2.67) to 5.16 per 100,000 in 2010 (95% CI: 4.85–5.47); a 2.1-fold increase (*p* < 0.05 for trend). A greater part of this increase is attributable to increase in incidence of papillary thyroid cancer, which increased from 1.64 to 3.61 per 100,000; a 2.2-fold increase (p < 0.05 for trend). Follicular cancer showed lesser, yet a significant increase from 0.56 to 0.95 per 100,000 (*p* < 0.05). Other varieties of thyroid cancer showed no significant increases in incidence. Trends in the increases in incidence of papillary cancer in females showed a much greater increase compared with males (from 2.45 to 5.60 per 100,000, a 2.28-fold increase in females compared with from 0.82 to 1.55; a 1.89-fold increase in males, *p* < 0.001). Highest incidence of papillary cancer was observed in 30–39-year age group, which has increased from 5.56 to 12.9 per 100,000; a 2.32-fold increase (*p* < 0.001).

**Conclusions:**

The increasing incidence of thyroid cancer in Sri Lanka is predominantly due to the increasing incidence of papillary cancers. These trends may reflect increased detection and better reporting, although an inherent increase in the incidence is the likely main contributor. Further studies including tumour stage and mortality may help answer these questions.

## Background

Thyroid cancer is a common cancer in both developed and developing countries, and is currently the 3rd most common cancer among Sri Lankan women [1]. In many developed countries including the United States [2], Canada [3] and Australia [4], the incidence of thyroid cancer has more than doubled over last two to three decades. It remains the most common endocrine malignancy contributing to approximately 2.5% of all cancers in these countries [5]. Since the latter part of the last century, it has become the fastest increasing cancer among women in developed countries. In contrast, incidence of many other neoplasms including lung, colon, and breast has observed a decreasing trend [6].

While the trends and patterns in thyroid cancer incidence are studied in detail in developed countries, analyses of these trends and patterns have been performed in developing countries only occasionally. Regardless, available evidence from developing and countries in the Asian region suggest that increases in incidence is perhaps greater than that of Western countries [7]. For instance, a four-fold increase in thyroid cancer is reported from Shanghai, China over a 25-year period from 1983 to 2007 [8].

Some researchers have proposed that the increasing incidence of thyroid cancer may be an artefact due to improved sensitivity of diagnostic tests and better reporting of cases rather than representing a true increase in the occurrence of thyroid cancer [2, 9]. However, others argue that increased detection through improved diagnostic technologies cannot fully explain the observed increase [10–13]. For instance, a US study based on surveillance epidemiology and end results (SEER) data found an increase in tumours > 4 cm and tumours diagnosed at an advanced stage, both of which are likely to be symptomatic at the time of diagnoses [10]. Current evidence from developed countries suggest that both an increase in true incidence and increased diagnostic scrutiny are responsible for the observed increase, although the extents of these contributions are unclear [14].

Irrespective of the underlying reasons, increasing incidence of a cancer has significant implications to a country’s healthcare system and allocation of resources. The healthcare and economic impact of thyroid cancer have generally been under-appreciated compared with other common malignancies, for instance breast and colorectal cancer [15]. Thyroid cancers are primarily treated with surgery while radioactive iodine therapy is the main adjuvant therapy. Both surgical facilities and radioactive iodine are in short supply in many developing countries [16]. Hence understanding the trends of thyroid cancer may help these resource limited countries to plan and utilize resources more effectively.

Sri Lanka National Cancer Control Programme (NCCP) has been collecting nationwide cancer data since 1985. Over the last 30-year period, the coverage has gradually increased and as of 2014, it is estimated to include well over 80% of all cancers diagnosed in the country [1]. NCCP data include all cancers treated at national cancer treatment centres in addition to data from other major government and private hospitals and pathology laboratories. As adjuvant treatments are instituted almost exclusively through cancer treatment centres, the coverage for thyroid cancers are likely to be greater than the overall coverage of 80%.

This study was conducted to identify recent trends in the incidence of thyroid malignancies in Sri Lanka based on data from the NCCP, and to discuss in relation to other regional and international thyroid cancer data. We also intended to assess age specific, gender-specific and histopathological subtype specific thyroid cancer incidence rates.

## Methods

### Study population

Cancer related details of all thyroid cancer patients diagnosed between 01/01/2001 and 31/12/2010 were extracted from the publications of cancer incidence data of Sri Lanka by the National Cancer Control Programme [1]. The classification of thyroid cancer was based on ICD-10 system (C73).

### Statistical analysis

Age standardized rates of thyroid cancer per 100,000 population were calculated for each year by gender and histology subtype, using WHO age standardized populations [17]. Moreover, age group specific rates were calculated for each year under consideration. Age group categories were selected as < 20, 20–49, 40–59 and 60+ years.

Joinpoint regression analysis was used to identify points where a statistically significant change over time in linear slope of the trend occurred [18]. This analysis starts with the minimum number of joinpoints, and tests whether one or more joinpoints are statistically significant, and should be added to the model. Joinpoint tests of significance use a Monte Carlo permutation method [19]. In the final model, each joinpoint indicates a statistically significant change in trend, and an estimated annual percentage change (EAPC) computed for each of those trends by means of generalized linear models assuming a Poisson distribution. Changes in direction or in the rate of increase or decrease were calculated with *p* values and p values < 0.05 were considered as statistically significant. Joinpoint software version 4.3 was used for Joinpoint regression analysis.

## Results

This study included a total of 7681 thyroid malignancies diagnosed over the 10-year study period. The commonest histological type was papillary (*n* = 5302, 69%) followed by follicular (*n* = 1411, 18.4%), medullary (*n* = 138, 1.8%), anaplastic (*n* = 285, 3.71%) and other rarer varieties (*n* = 545, 7.1%). The majority of the cancers were in females (*n* = 6166, 80.3%) with a male to female ratio of 1: 4.07. Mean age of study patients was 43.8 years. Male patients were significantly older at diagnosis compared with female patients (mean age: 47.4 versus 42.9 years, respectively, *p* < 0.001). Highest incidence of thyroid cancers was observed in 30–39 age group, overall (6.1 per 100,000 population) and for female and male groups separately (10.2 and 1.9 per 100,000 population, respectively).

**Fig. 1:**
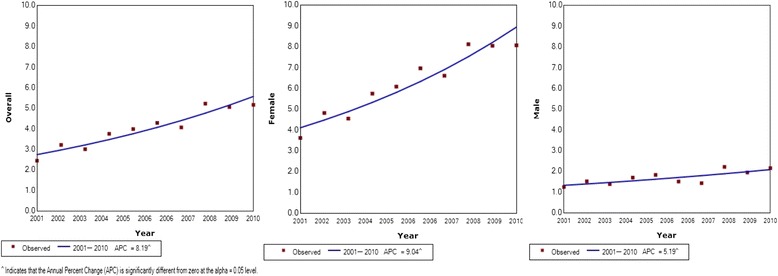
Joinpoint regression curve for age standardized incidence rates for thyroid cancer per 100,000 population in Sri Lanka 2001–2010

Results of thyroid cancer incidence in Sri Lanka with Joinpoint analysis of trends by gender, age group and histology subtype are shown in Table 1. The WHO age standardized incidence of thyroid cancer in Sri Lanka was observed to have increased significantly from 2.44 per 100,000 in 2001 (95% confidence interval [95% CI]: 2.21–2.67) to 5.16 per 100,000 in 2010 (95% CI: 4.85–5.47); a 2.1-fold increase (*p* < 0.05 for trend) (Fig. 1). This increase translates into an estimated annual percentage change (EAPC) of 8.2 (95% CI 5.9–10.5) (Table 1). The proportional increase in incidence was greater for females (from 3.61 to 8.06; a 2.23-fold increase, *p* < 0.05 for trend) compared with males (from 1.24 to 2.15; a 1.73-fold increase, p < 0.05 for trend). Further, cancer incidence in females appears to have increased exponentially while in males it has been increasing at a steady rate (Fig. 1).

**Table 1:** Thyroid cancer incidence in Sri Lanka by gender, age group and histology subtype with Joinpoint analysis 2001–2010

	2001		2010		EAPC^a^ 2001–2010 (95% CI)
	n	Rate (95% CI)	n	Rate (95% CI)	
Age group (years) Male					
< 20	1	0.03	7	0.19	16.2 (−1.7–37.4)
20–39	39	1.29	61	1.84	4.2 (0.0–8.6)
40–59	40	1.93	83	3.66	5.9 (0.2–11.9)
60+	26	3.16	52	5.77	5.3 (0.6–10.2)
Age standardized	106	1.24 (1.00–1.47)	203	2.15 (1.85–2.44)	5.3 (1.9–8.8)
Age group (years) Female					
< 20	13	0.39	21	0.57	5.3 (0.4–10.5)
20–39	143	4.66	363	10.7	9.8 (7.2–12.6)
40–59	127	5.95	348	14.8	9.2 (6.3–12.2)
60+	54	5.90	99	9.81	4.1 (0.8–8.5)
Age standardized	337	3.61 (3.23–4.00)	831	8.06 (7.52–8.65)	9.0 (6.6–11.5)
Overall age standardised rate	443	2.44 (2.21–2.67)	1034	5.16 (4.85–5.47)	8.2 (5.9–10.5)
Histology type (Male)					
Papillary	72	0.82 (0.63–1.01)	149	1.55 (1.32–1.79)	7.0 (2.9–11.2)
Follicular	19	0.22 (0.12–0.32)	23	0.25 (0.15–0.35)	−0.2 (− 2.7–2.5)
Other	15	0.23 (0.12–0.35)	31	0.35 (0.23–0.47)	3.5 (−2.4–9.8)
Histology type (Female)					
Papillary	232	2.45 (2.13–2.82)	586	5.64 (5.11–6.13)	9.3 (7.0–11.8)
Follicular	81	0.94 (0.74–1.12)	165	1.62 (1.42–1.93)	8.0 (5.6–10.5)
Other	24	0.32 (0.22–0.45)	80	0.80 (0.63–1.02)	9.0 (1.7–16.8)
Histology type (Overall)					
Papillary	304	1.64 (1.46–1.83)	735	3.61 (3.35–3.87)	8.8 (6.6–11.1)
Follicular	100	0.56 (0.45–0.67)	188	0.95 (0.82–1.09)	6.5 (5.2–7.9)
Other	39	0.24 (0.16–0.31)	111	0.61 (0.49–0.71)	7.6 (1.0–14.6)

^a^*EAPC* Estimated Annual Percentage Change

**Fig. 2:**
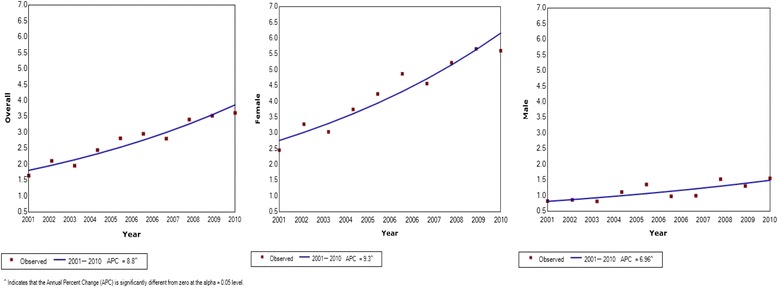
Joinpoint regression curve for age standardized incidence rates for papillary thyroid cancer per 100,000 population in Sri Lanka 2001–2010

A greater part of the increase in thyroid cancer incidence is attributable to an increase in incidence of papillary thyroid cancer, which has increased from 1.64 to 3.61 per 100,000; a 2.2-fold increase (p < 0.05 for trend) (Fig. 2). Trends in the increases in incidence of papillary cancer was found to be similar to the overall increase in thyroid cancer with females showing a much greater increase compared with males (from 2.45 to 5.60 per 100,000, a 2.28-fold increase in females compared with from 0.82 to 1.55; a 1.89-fold increase in males, *p* < 0.001). Highest incidence of papillary cancer was observed in 30 to 39-year age group, which has increased from 5.56 to 12.9 per 100,000; a 2.32-fold increase over the 10-year study period.

Follicular cancer showed a much smaller increase from 0.56 to 0.95 per 100,000 (a 1.59-fold increase; *p* < 0.05 for trend) compared with papillary cancer. Increase in follicular cancer incidence was much greater and significant for females (from 0.9 to 1.6 per 100,000 population; a 1.8-fold increase, p < 0.05) while for males the incidence has essentially remained static over the study period (from 0.22 to 0.25 per 100,000 population, a 1.1-fold increase, *p* > 0.05 for trend). Other less common varieties of thyroid cancer incidence showed no substantial increases over this period.

**Fig. 3:**
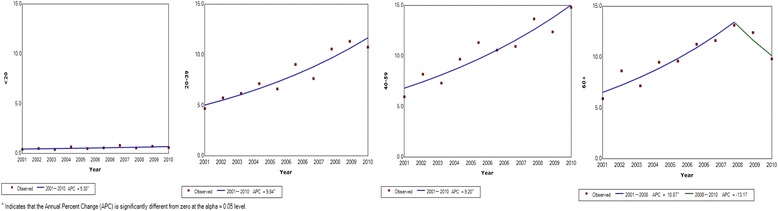
Joinpoint regression curve for thyroid cancer incidence by age category in females per 100,000 population in Sri Lanka 2001–2010

**Fig. 4:**
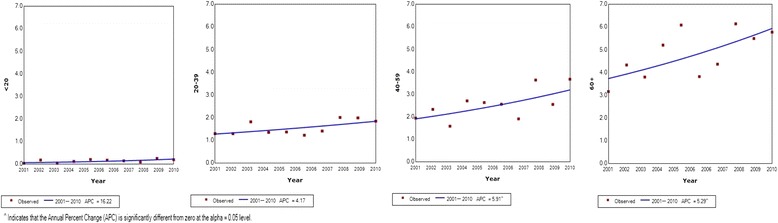
Joinpoint regression curve for thyroid cancer incidence by age category in males per 100,000 population in Sri Lanka 2001–2010

Incidence of thyroid cancer peaked in 40–59 age group in females while in males a gradual increase in the incidence with age was observed with the highest incidence observed among above 60-year age group (Figs. 3 and 4). A substantial reduction in thyroid cancer incidence among women over 60 years was observed over last 2 years of the study which was not seen among males.

## Discussion

In Sri Lanka, as in many other developed and developing countries, the incidence of thyroid cancer appears to be increasing at a substantial rate. Most likely, these trends reflect an inherent increase in incidence which possibly is augmented by better diagnostic scrutiny and improvements in cancer recording and reporting.

There are many well-documented risk factors in the development of thyroid cancer. These include exposure to ionizing radiation [20], iodine deficiency [21] auto immune thyroiditis [22] and long-standing multi nodular goitres (MNG) [23], while there are other risk factors which are less clear including diet, body weight [22], insulin resistance and exposure to environmental pollutants [24].

Exposure to ionizing radiation is unlikely to be an important factor in Sri Lanka as either industrial or medical exposures. First, Sri Lanka or neighbouring parts of India does not have nuclear reactors. Second, radiotherapy facilities have been limited to a handful of cancer treatment centres in Sri Lanka which hence have prioritized malignant over non-malignant conditions for provision of radiotherapy [25]. Although individual radiation exposures have increased in the recent past due to medical exposures, for instance imaging investigations, as thyroid cancer is a disease of the relatively young who unlikely to have had a significant medical radiation exposures, makes it unlikely to be an important factor. National salt iodization programme started in early 1990s has virtually eradicated iodine deficiency in Sri Lanka and is expected to have a protective effect on thyroid cancer [26]. Although the incidence of MNG has decreased substantially with the introduction of salt iodization programme, the incidence of MNG still remains high at approximately 6.8% [27]. The declining incidence of MNG would have expected to have led to a reduction in the incidence of thyroid cancer and not an increase as observed in this study [28]. On the other hand, evidence from Sri Lanka and other countries suggests that the incidence of chronic thyroiditis has been on the rise in parallel with salt iodization [29] which is a well-recognized risk factor for differentiated thyroid cancer [22]. Previous Sri Lankan studies have shown a gradual increase in the presence of thyroid peroxidase auto antibodies among school age girls since the implementation of the national salt iodization programme [30]. This may be a possible reason for the observed increase in thyroid cancer although the effect size is unlikely to fully explain the observed increase.

Of the lesser known risk factors, obesity, diet including increasing use of processed food and insulin resistance have been increasing rapidly in the country [31]. Although there are many environmental pollutants which potentially increase the risk of thyroid cancer including cigarette smoke, asbestos, benzene, formaldehyde and pesticides, their direct correlation has not been ascertained yet. Regardless, based on epidemiological data, the associations appear to be relatively weak and unlikely to have played a major role in the observed increase in incidence [32].

The increase in diagnostic scrutiny and changes in practice have been suspected to be of aetiological importance in the observed increase in thyroid cancer incidence. For instance, a recent introduction of a nationwide thyroid cancer screening programme in South Korea has seen a 10-fold increase in the incidence of thyroid cancer over a period of 10 years [33]. Although there are no national consensus guidelines on management of thyroid nodules or goitres, Sri Lanka has closely followed guidelines from Western countries in subjecting almost all patients presenting with thyroid nodules for ultrasonography and fine-needle aspiration cytology (FNAC). As ultrasonic evaluations of the neck have become commonplace, both for assessment and as a screening modality for thyroid nodules, many tumours which otherwise would not have been diagnosed are picked up and treated. Available limited data from Sri Lanka suggest that more thyroid cancers are diagnosed at early stages which probably reflect improving health literacy and healthcare access, although some have suggested salt iodization programme has been responsible for these changes [34].

Thyroid carcinomas are well-known to occur without ever clinically manifesting during the life. In a review of autopsy studies published between year 1952 and 1998, Arem et al. reported that the prevalence of thyroid carcinomas ≤1.5 cm among people who died of unrelated causes to be 5–10% [35]. In some regions, the prevalence is higher than others with the highest reported occult carcinoma being reported from Finland which is 36% [36]. These findings, together with the stable thyroid cancer mortality of < 1% per 100,000 individuals per year, support the hypothesis that the increase in incidence may be more likely an artefact of improved detection.

However, proponents of a true increase in incidence argue that the stable mortality rates are due to earlier detection and better treatment of these thyroid cancers which otherwise would have had a poor outcome. A birth cohort analysis has suggested that increased environmental exposures might have contributed to the observed increase during the past three decades [13], which also supports the hypothesis of a true increase. Current study has also shown some evidence to support a true increase at least among some groups. For instance, the incidence of follicular carcinoma in males has remained unchanged (< 10% increase) while in females it has increased by about 80%. This may reflect differential action of risk factors contributing to a greater increase of follicular carcinoma in females. Further, a decrease in incidence was observed among women older than 60 years over the last 3 years of the study. If the increase in incidence observed in this study was due to better diagnosis and reporting, a similar increase would have been seen in this group. While it is unclear why the incidence is declining in this group, the declining incidence indirectly support the notion that there has been a true increase in thyroid cancer incidence among other age and gender groups.

The data presented in this study does not allow for differentiation between a true increase in incidence versus a better diagnosis and reporting. It is likely that both these factors have been contributory to different degrees. Regardless of the aetiology, the increase in incidence and patterns of increase shown here have important implications. As most of the cancer care for thyroid cancer is provided through the public sector, the increasing incidence has caused a major strain on the availability of cancer care services. For instance, wait lists extending well over a year are not unusual especially for the delivery of radioactive iodine therapy in the public sector [37]. This is further compounded by the low threshold in recommending radioiodine therapy for low and intermediate risk thyroid cancer observed among oncologists’ due to uncertainties in patient follow up. Healthcare policy makers need to consider all these factors to increase the availability and the efficiency of thyroid cancer care to reduce wait times which may lead to suboptimal outcomes and cause unnecessary psychological stressors for patients.

This study has several limitations. First, the coverage of cancer data has changed over the study period complicating the interpretation of study findings. Further, unavailability of good data on cancer stage at diagnosis and cancer specific mortality prevented us from performing further analyses to identify the potential contributions of increased diagnosis versus a true increase in incidence towards the observed increase in incidence [10, 13]. Regardless, NCCP data constitutes the largest and most comprehensive database on thyroid cancer in Sri Lanka. While acknowledging above limitations, these findings are likely to be the most accurate in relation to patterns in thyroid cancer in Sri Lanka.

## Conclusions

This study has shown the incidence of thyroid cancer in Sri Lanka to be on the rise. Although it is uncertain whether this is due to better diagnosis and reporting or due to a true increase, increasing numbers clearly have resulted in long wait lists for cancer care. While further studies are needed to examine the reasons for observed increase in incidence, strategies are needed to be implemented as a matter of urgency to improve cancer care services for thyroid cancer patients to minimize current delays.

## Abbreviations

CI: Confidence interval
EAPC: Estimated annual percentage change
MNG: Multinodular goitre
NCCP: National Cancer Control Programme
WHO: World Health Organization
